# Fat Absorption after Gastrojejunostomy

**Published:** 1908-07-11

**Authors:** 


### FAT ABSORPTION AFTER GASTROJEJUNOSTOMY.

When it is remembered that Pawlow proved that the strongest stimulus to the secretion of the pancreatic juice is the passage of acid chyme through the pylorus into the first part of the duodenum; that the juices which are most potent in the digestion of fats are the bile and the pancreatic juice together; and that when gastrojejunostomy has been performed it is probable that much of the gastric content passes directly into the jejunum without going into the duodenum at all: it might be thought that gastrojejunostomy must inevitably remove a great part of the normal stimulation of the biliary and pancreatic secretions, with the result that fat absorption must thereafter be deficient. This is a point which could only be settled by direct and special investigation, and the profession cannot but be grateful to Dr. H. C. Cameron for the work he has done upon the subject at Guy's Hospital. If confirmed, his results prove that patients who have had gastrojejunostomy performed for non-malignant conditions of the stomach, notably pyloric obstruction from cicatrisation of a simple ulcer, are able to take as much fat as healthy persons do, and absorb a normal proportion of the whole of it. This result is of interest in more ways than one. In the first place it is a very convincing example of the necessity of never accepting theoretical deductions without first putting them to the test of practical investigation; in the instance under discussion the actual results are quite contrary to the theoretical expectations. In the second place, Dr. Cameron's work removes what might have been a serious objection to the operation. If it had turned out that these patients were rendered unable to assimilate fats as the result of the operation, the latter would naturally have been postponed in many cases in which the pylorus was not so stenosed as to make surgical assistance an absolute necessity for the stomach. Things being as they are, on the contrary, the fact that the operation does not interfere with the absorption of fats, nor indeed with the digestion and absorption of proteids either, is an additional reason for having it done in cases in which purely medical treatment is unsuccessful. In the third place, it is important to know that there need not be any special restriction in regard to the amount of fats these patients take after the operation; if a large proportion of the fat had been found to pass out with the fæces, it might have been necessary to restrict the amount ingested, for fear of rancid and putrefactive changes occurring in the residue that was unabsorbed from the bowel; necessity for such restriction disappears, except in so far as the tastes and idiosyncrasies of different persons as regards their liking or disliking of fat go.

It is needless to give more than three examples of Dr. Cameron's cases. The diet consisted of an abundance of mixed foods, including 3 ounces of butter daily, milk, cream, eggs, cod, haddock, lean meat, bread, vegetables, and weak tea with sugar. The following figures speak for themselves:—

| | Total fat in food per diem | Total fat in fæces per diem | Percentage of fat absorbed |
|---|---|---|---|
| | grams | grams | per cent. |
| Female, aged 43 ... ... Gastrojejunostomy for severe stenosis of pylorus by old ulcer; operation about 18 days previously. | 122.49 | 5.25 | 95.71 |
| Male, aged 41 ... ... A similar constricted pylorus; gastrojejunostomy a fortnight previously. | 201.3 | 7.00 | 96.57 |
| Male, aged 68 ... ... Gastrojejunostomy 8 years previously; fibrous stricture of pylorus. | 126.54 | 8.07 | 93.60 |

These figures are practically identical with those of health, except in regard to the fact that the patients were purposely being given a good deal more fat than most persons eat; notwithstanding this they absorbed about 95 per cent. of it, and this is to all intents and purposes identical with the proportion of ingested fat that is absorbed by normal persons.

